# Comparing Utility of Intraoperative Magnetic Resonance Imaging and 5-Aminolevulinic Acid in High-Grade Glioma Resection Surgery: A Systematic Review and Meta-Analysis

**DOI:** 10.1227/neuprac.0000000000000146

**Published:** 2025-06-27

**Authors:** Nova Kristine de los Reyes-Nabhan, Siddharth Sinha, Imran Noorani

**Affiliations:** * Division of Neurosurgery, National Hospital for Neurology and Neurosurgery, London, UK;; ‡ Department of Neurosurgery, Sana Klinikum Offenbach, Academic Teaching Hospital of Johann Wolfgang Goethe-University, Frankfurt am Main, Germany;; § Department of Neuromuscular Diseases, Institute of Neurology, UCL, London, UK

**Keywords:** Intraoperative MRI, 5-aminolevulinic acid, Gliolan, Glioblastoma, High-grade glioma, Resection surgery, Extent of resection

## Abstract

**BACKGROUND AND OBJECTIVES:**

High-grade glioma has a poor prognosis despite advancements in histopathological classifications and treatments. Various intraoperative modalities are used to maximize extent of resection (EoR) and intraoperative detection of residual tumor, including 5-aminolevulinic acid (5-ALA) and intraoperative MRI (iMRI). We conducted a systematic review with meta-analysis investigating the efficacy of iMRI vs 5-ALA in maximizing EoR and improving survival outcomes.

**METHODS:**

A systematic review with meta-analysis was performed using Preferred Reporting Items for Systematic Reviews and Meta-Analysis guidelines, PubMed, Embase, Scopus, and ClinicalTrials.gov databases, identifying randomized clinical trials (RCTs) and prospective studies comparing the use of 5-ALA and iMRI in high-grade glioma resection. The primary end points were EoR and survival outcomes. Quality assessment was conducted using the ROBINS-I risk of bias assessment and Jadad scale. Meta-analysis was performed using gross total resection rates, tumor detection sensitivity, and specificity.

**RESULTS:**

Five RCTs and 5 prospective studies were identified. Five RCTs lacked published data, thus only 5 prospective studies were included in the data extraction. Combined 5-ALA with iMRI (100%) was superior to 5-ALA alone (61.7%; *P* < .002) in maximizing EoR. Gross total resection did not differ significantly between 5-ALA alone (78%) and iMRI alone (81%; *P* = .79). One study showed that specificity was higher with iMRI alone (0.70) than with 5-ALA alone (0.43; *P* < .001); however, this was not replicated by 2 other studies (iMRI vs 5-ALA: 0.60 vs 0.80, *P* < .001; 1.00 vs 1.00, *P* not significant). Two studies reported sensitivity; only 1 found lower sensitivity with iMRI vs 5-ALA with a significant difference (iMRI vs 5-ALA: 0.66 vs 0.90, *P* < .001).

**CONCLUSION:**

There is no clear evidence to suggest iMRI is superior to 5-ALA in maximizing EoR and improving survival. However, combined use of 5-ALA and iMRI may be more effective compared with either modality alone. Larger RCTs are needed to confirm any differences in efficacy between the 2 modalities.

**ABBREVIATIONS:**
5-ALA: 5-aminolevulinic acid
EoR: extent of resection
GTR: gross total resection
HGG: high-grade glioma
iMRI: intraoperative MRI
RCT: randomized clinical trial.

High-grade gliomas (HGGs) are the most prevalent primary intrinsic brain malignancies.^1^ Despite developments in histopathological classifications and treatments,^2,3^ prognosis of these patients remains poor with a median overall survival of 15 to 18 months.^1,3^ Extent of resection (EoR) and gross total resection (GTR) are favorable prognosticators in these patients.^4-6^ New molecular classifications suggest a survival benefit in maximizing the EoR in newly diagnosed and recurrent glioblastomas of the DNA methylation subclasses RTK1 and RTK2.^7,8^

Various intraoperative adjuncts have been used to maximize EoR in HGG surgery, including use of neuronavigation, ultrasound,^9^ 5-aminolevulinic acid (5-ALA),^10,11^ and intraoperative MRI (iMRI). Although iMRI was introduced more than 2 decades ago, its utilization remains limited to specialized centers in developed countries, with running costs, additional time added to surgery, and equipment procurement being limiting factors in its widespread use in neurosurgery.^12-14^

Owing to its ability to provide high-resolution images of the intraoperative site during resection surgery and thus identify residual tumor through contrast-enhancing areas, there is a prevalent assumption that iMRI might be superior to 5-ALA in achieving GTR and improving survival outcomes in this patient population.^12,15,16^ Some studies have suggested there is an added benefit of iMRI to conventional resection surgery of glioblastoma using neuronavigation.^12,13,15^ However, a systematic review from 2011 investigating iMRI vs neuronavigation in glioblastoma surgery found limited evidence to suggest iMRI was more effective than neuronavigation in maximizing EoR and improving outcomes.^12^ A meta-analysis of 1847 patients from 11 studies found that the addition of iMRI to neuronavigation significantly increased the EoR but did not result in significant improvement in overall survival or progression-free survival.^13^ A network meta-analysis including retrospective and prospective studies compared iMRI vs 5-ALA indirectly and found no difference in GTR.^17^ To date, a systematic review and meta-analysis of prospective studies and randomized controlled trials (RCTs) investigating the advantages of iMRI over 5-ALA in HGG resection surgery has been lacking.

The aim of this systematic review and meta-analysis was to compare tumor detection and clinical outcomes with iMRI vs 5-ALA as an intraoperative modality in surgical resection of HGG.

## METHODS

**FIGURE 1.**
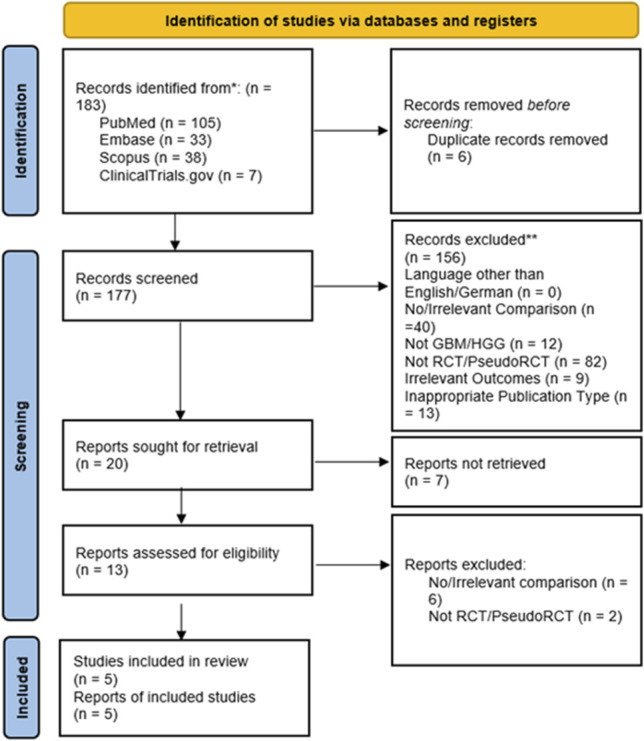
Preferred Reporting Items for Systematic Reviews and Meta-Analysis 2020 flow diagram for systematic review comparing use of 5-aminolevulinic acid and intraoperative MRI in HGG resection surgery. GBM, glioblastoma multiforme; HGG, high-grade glioma; RCT, randomized clinical trial.

We conducted a systematic review according to the Preferred Reporting Items for Systematic Reviews and Meta-Analysis guidelines^18^ (Figure 1, **Supplemental Digital Content 1** [http://links.lww.com/NS9/A52]). A search of the databases PubMed, Embase, and Scopus, including trial registry, ClinicalTrials.gov, was performed on 01 July, 2024, for journal articles comparing use of 5-ALA and iMRI in HGG resection surgery, especially targeting RCTs and prospective studies.

We used synonyms of the terms “5-ALA,” “iMRI,” “glioblastoma,” “high-grade glioma,” “resection surgery” and “randomized clinical trial” in the search terms (**Supplemental Digital Content 2** [http://links.lww.com/NS9/A53]). The search yielded a total of 183 results: 105 identified from PubMed, 33 from Embase, 38 from Scopus, and 7 from ClinicalTrials.gov. Using Zotero citation manager (Corporation for Digital Scholarship), 6 duplicates were identified and removed before screening.

Inclusion criteria for screening were (1) RCT or prospective studies, (2) studies comparing both iMRI and 5-ALA, and (3) studies on patients with HGG. Exclusion criteria were (1) studies that do not report on the primary outcomes of interest; (2) studies with insufficient or unclear data regarding outcomes; (3) publication types of abstracts, conference proceedings, editorials, commentaries, and letters to the editor, unpublished data, theses, or dissertations; (4) studies published in languages other than English and German; (5) retrospective studies; (6) studies investigating only iMRI or only 5-ALA or not comparing 5-ALA and iMRI; and (7) studies on patients with low-grade glioma.

After screening 177 records, 20 studies were sought for retrieval. The 7 RCTs registered in ClinicalTrials.gov were not retrieved as journal publications, leaving 13 studies for eligibility assessment. Of these, 2 studies were removed for not meeting the inclusion criterion of RCT/prospective studies, and 6 were removed for meeting the exclusion criterion of investigating only iMRI or only 5-ALA or not comparing 5-ALA and iMRI. Ultimately, 5 studies were included in the review.

Before data extraction, the review was registered at the PROSPERO International prospective register of systematic reviews. IRB approval and patient consent were waived because all 5 studies obtained these. We extracted the following data from each study: study authors, country, publication year, number of patients included in the study, percentage of female patients (sex ratio), age range of patients, follow-up time in months, study design, inclusion criteria, exclusion criteria, timeframe, comparison of interventions, primary end point, measures of primary end point, 5-ALA protocol used, iMRI device, iMRI sequences used, extra OR time, definition of GTR, tumor volumetry, confirmation of complete resections, assessment of EoR, limitations of 5-ALA alone and iMRI alone, advantages of iMRI, clinical performance, complication rates, progression-free and overall survival rates, and conclusions derived from each study. The results of the data extraction are presented in Tables 2-5.

After removing 2 RCTs not comparing 5-ALA and iMRI, 5 RCTs with unpublished results and/or are ongoing clinical trials are listed in Table 1. From these, relevant data such as trial center, registration year, study design, research question, and Population, Intervention, Comparison, and Outcome (PICO)-methodology as well as current trial status were noted.

**TABLE 1.** RCTs Comparing 5-ALA and iMRI in High-Grade Glioma Resection Surgery

Trial	ClinicalTrials.gov ID	Center	Registration year	Study design	Research question	Population	Intervention	Outcomes/end points	Current status
Combined Approach to Resection of Glioblastoma by 5-ALA and Intraoperative MRI	NCT01208909	University of Zurich, Switzerland	2010	Prospective, case-control	Can the combination of 5-ALA fluorescence and intraoperative MRI increase the number of sites where tumor tissue can be detected?	Patients with GBM	Diagnostic 5-ALA and iMRI for tumor resection	Number of sites of detected tumor tissue	Last updated 2010, ongoing
A Phase 2 Comparative Study of 5-ALA and iMRI to Enhance Completeness of Resection of Glioblastoma	NCI-2012-00452	Case Comprehensive Cancer Center, USA	2012	Prospective, interventional	Are there are differences in what Gliolan shows a surgeon compared with iMRI?	Adult giant cell glioblastoma, adult glioblastoma, adult gliosarcoma, recurrent adult brain tumor	5-ALA vs iMRI in intraoperative visualization of tumor	TDP, overall survival	Last updated 2018, completed
iMRI and 5-ALA Guidance to Improve the EoR in Brain Tumor Surgery (IMAGER)	NCT01798771	Johann Wolfgang Goethe University Hospital, Frankfurt, Germany	2013	Prospective, interventional, randomized	Is the rate of radiologically complete resections of contrast-enhancing brain tumors following surgeries aided by use of 5-ALA induced fluorescence guidance and use of an intraoperative ultra-low field MRI higher compared with surgeries aided by 5-ALA induced fluorescence alone?	Primary supratentorial intra-axial brain tumor exhibiting contrast enhancement suspected to be malignant glioma	5-ALA with iMRI vs 5-ALA alone in achieving GTR	EoR, volumetric EoR, PFS 6, PFS 12, quality of life, NIHSS	Last updated 2013, ongoing
Impact of iMRI on the EoR in Patients With Newly Diagnosed Glioblastomas—A Prospective Multicenter Parallel Group Clinical Trial	NCT02379572	University Hospital Tübingen, Germany	2015	Prospective, interventional, randomized, parallel-group approach	What is the value of iMRI guidance in the resection of GBMs in comparison with conventional 5-ALA microsurgery?	In MRI suspected primary singular untreated GBM	iMRI-guided surgery vs 5-ALA-guided surgery	Number of total resections (no residual contrast enhancement) in the postoperative MRI (T1 + CM within 48 h after surgery) in each group; perioperative clinical data, progression free survival, patients' clinical condition, and overall survival	Last updated 2021, ongoing
MR Fingerprinting Guided Extended Resection in Glioblastomas	NCT06455189	Case Comprehensive Cancer Center, USA	2024	Prospective, interventional, randomized, parallel-group approach	Can MR fingerprinting, a new way of acquiring MRI images, help identify the extent of tumor spread in the brain, better than routine MRI images?	Patients with MR imaging findings suggestive of glioblastoma	5-ALA vs intraoperative MRF/MRI infiltration guidance for extended resection	SAEs at 48 h and 30 d post-targeted biopsy sampling procedure. Feasibility as assessed by the performance of MRF/MRI infiltration mapping guidance in surgical resection of new glioblastomas; PFS, EoR, operator confidence, histopathological correlation, recurrence	Last updated 2024, ongoing

5-ALA, 5-aminolevulinic acid; CM, contrast medium; EoR, EoR, extent of resection; GBM, glioblastoma multiforme; IMAGER, iMRI and 5-ALA Guidance to Improve the EoR in Brain Tumor Surgery; iMRI, intraoperative MRI; MRF, magnetic resonance fingerprinting; NIHSS, National Institutes of Health Stroke Scale; PFS, progression-free survival; TDP, time to disease progression.

Quality assessment of included studies was conducted using the Risk Of Bias In Non-randomised Studies-of Interventions (ROBINS-I) risk of bias assessment.^19^ The 1 RCT included was assessed using the widely used Jadad scale.^20^ Thematic analysis was performed using the tabulated results from the data extraction.

**FIGURE 2.**
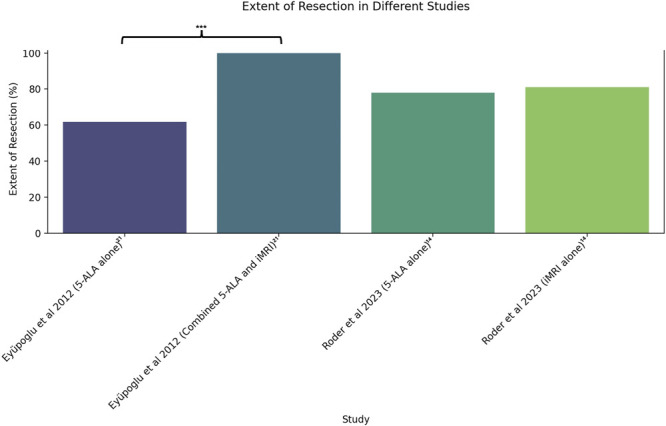
Extent of tumor resection (%) with 5-ALA and iMRI across Eyüpoglu et al^21^ and Roder et al.^14^ 5-ALA, 5-aminolevulinic acid; iMRI, intraoperative MRI.

**FIGURE 3.**
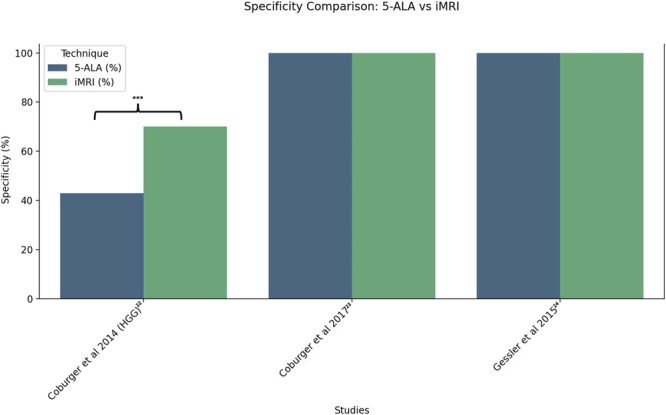
Specificity for tumor detection for 5-ALA and iMRI across 3 studies; Coburger et al^22,23^ and Gessler et al.^24^ 5-ALA, 5-aminolevulinic acid; iMRI, intraoperative MRI.

**FIGURE 4.**
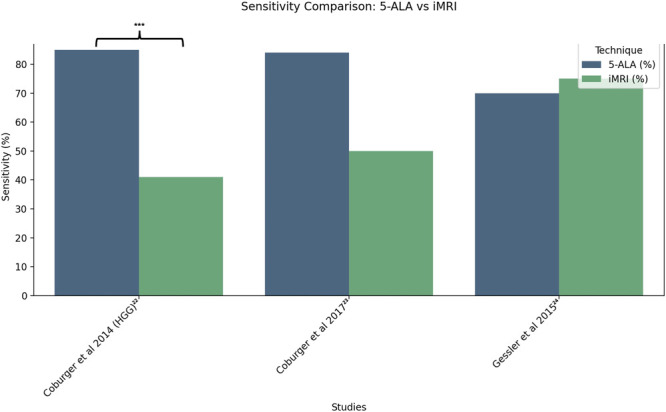
Sensitivity for tumor detection for 5-ALA and iMRI across 3 studies; Coburger et al^22,23^ and Gessler et al.^24^ 5-ALA, 5-aminolevulinic acid; iMRI, intraoperative MRI.

A meta-analysis was conducted for the 5 included studies, comparing either EoR or tumor detection sensitivity and specificity. First, EoR was compared between Eyüpoglu et al^21^ and Roder et al.^14^ Following this, tumor detection sensitivity and specificity was compared across 3 studies, Coburger et al,^22^ Coburger et al,^23^ and Gessler et al.^24^ The results of these comparisons are represented in Figures 2-4 using R, highlighting those with a significant difference in imaging modalities (*P* < .05).

## RESULTS

### Trials Summary

Our review yielded 5 RCTs, registered between 2010 and 2024, 4 of them ongoing (Table 1). One RCT was completed in 2018; however, results have yet to be published. Four RCTs compare iMRI vs 5-ALA during resection surgery of glioblastoma while 1 RCT investigates the combined use of iMRI and 5-ALA in increasing the number of detected sites with pathologic tissue intraoperatively. Three RCTs identify as their end points EoR and/or GTR rates, and rates of detecting tumor tissue. Four RCTs include rates of overall survival and progression-free survival as one of their end points or investigated outcomes. Owing to lack of published data from these 5 RCTs, they could not be included in the data extraction.

### Studies Summary

Four studies had a study size of 32-37^21-24^; the nonrandomized controlled trial investigated 314 patients^14^ (Table 2). Follow-up time was not specified in 3 of the studies,^21-23^ whereas 2 studies had follow-up time of 12 months (ie 48 weeks)^14^ and a median of 70 weeks^24^. Two studies investigated HGG (ie malignant gliomas WHO grade 3-4 including glioblastoma)^21,22,24^ and 3 studies glioblastoma only,^14,23^ in adult patients, with an age range of 32 to 82 years. Furthermore, 1 study compared both modalities with linear array intraoperative ultrasound (lioUS) in detecting residual tumor intraoperatively.^23^ The studies incorporated surgeries conducted between April 2009 and June 2020; however, 1 study did not specify timeframe of the study.^24^

**TABLE 2.** Included Studies and Their Study Design, PICO Criteria, Assessment of Bias

Study	Country	n	F %	Age, y	Follow-up time (mo)	Study design	Inclusion criteria	Exclusion criteria	Timeframe	Comparison	Primary end point	Measures of primary end point	Secondary end points	Jadad score
Eyüpoglu et al^21^	Germany	37	29.7	33-75	NS	Prospective, nonrandomized study	Malignant gliomas WHO grade 3-4 according to radiological appearance	Karnofsky Index < 70 points, medication with thrombocyte aggregation inhibitors	April 2009-April 2012	5-ALA resection alone vs 5-ALA resection followed by iMRI for verification of EoR and detection of contrast-enhancing residual tumor	Postoperative clinical assessment, EoR	Karnofsky Performance Scale Index, histological confirmation of resected tumor detected by iMRI but missed by 5-ALA	NS	Not applicable since not RCT
Coburger et al^22^	Germany	34	40.0	32-82	NS	Prospective, nonrandomized study	Planned GTR of HGG, no contraindication for 5-ALA or iMRI, informed patient consent	18 < patient age < 75	July 2012-July 2013	5-ALA vs iMRI in detection of invasive tumor at border zone of GBM	Detection of residual tumor confirmed by histopathological results of biopsied areas with 5-ALA fluorescence and with contrast enhancement in iMRI	Sensitivity and specificity of tumor detection, histology is used for residual tumor confirmation	NS	Not applicable since not RCT
Gessler et al^24^	Germany	32	34.4	32-76	Median follow-up 70 wk (21.4-170.6 wk)	Prospective, nonrandomized study	Patients harboring malignant gliomas undergoing intended GTR	Intended subtotal resection and histology other than malignant glioma	NS	5-ALA vs iMRI in detection of residual tumor not visible in white light	Sensitivity and specificity of iMRI and 5-ALA fluorescence to detect remaining tumor tissue, confirmed by histology	Sensitivity and specificity of iMRI and 5-ALA fluorescence to detect remaining tumor tissue	Complication rates, progression-free survival, overall survival, KPS	Not applicable since not RCT
Coburger et al^23^	Germany	33	43.8	33-78	NS	Prospective, nonrandomized study	Patients older than 18 y with an intended GTR, and final histopathological assessment of a primary glioblastoma WHO grade 4	Patients with recurrent surgery or prior radiation	May 2012-October 2013	5-ALA vs iMRI vs lioUS in detecting residual tumor	Sensitivity and specificity	Sensitivity and specificity	NS	Not applicable since not RCT
Roder et al^14^	Germany	277	37.2	53-71	Follow-up at 3, 6, 9, and 12 mo with MRI, clinical assessment, RANO criteria evaluation, KPS, NIHSS, and EORTC QLQ-C30 and QLQ-BN20	Prospective, nonrandomized, controlled, multicenter clinical trial	Patients 18-80 y with suspected glioblastoma based on MRI, a planned complete resection of contrast enhancing tumor, and a KPS of ≥60%	Tumors of the midline, basal ganglia, cerebellum, brainstem, eloquent areas, multifocal glioblastoma, >50% noncontrast enhancing tumor, no informed consent, increased thrombosis risk, pregnancy or breastfeeding, 5-ALA hypersensibility, renal or hepatic insufficiency, and inability to receive adjuvant therapy, final histologic result deviating from glioblastoma WHO grade 4	July 2015-June 2020	5-ALA alone vs iMRI alone	Complete resections, defined as a residual contrast-enhancing tumor ≤0.175 cm on postoperative MRI within 48 h after surgery, evaluated by the central blinded neuroradiology reference	Resectability and EoR by an independent blinded centralized review of preoperative and postoperative MRI with 1-mm slices, KPS, NIHSS, and EORTC QLQ-C30 and QLQ-BN20	Preoperative and postoperative tumor volumes (cm^3^) and progression according to MRI using RANO criteria (imaging criteria only; clinical criteria were rated locally), PFS, OS, clinical data, histology, IDH-1, MGMT promoter status, and QoL	3

5-ALA, 5-aminolevulinic acid; EoR, EoR, extent of resection; EORTC, European Organisation for Research and Treatment of Cancer; GBM, glioblastoma multiforme; GTR, gross total resection; HGG, high-grade glioma; IDH-1, isocitrate dehydrogenase-1; iMRI, intraoperative MRI; KPS, Karnofsky performance score; lioUS, inear array intraoperative ultrasound; MGMT, methylguanine-DNA-methyltransferase; NIHSS, National Institutes of Health Stroke Scale; NS, not specified; OS, overall survival; PICO, Population, Intervention, Comparison, and Outcome; RANO, Response Assessment in Neuro-Oncology; RCT, randomized clinical trial.

All studies used histopathological confirmation of presence of pathologic tissue as gold standard against which interventions, ie 5-ALA or iMRI, were measured. Four studies had as their primary end point sensitivity and specificity of each modality (5-ALA and/or iMRI) in detecting residual tumor intraoperatively. Eyüpoglu et al^21^ investigated specifically the rates of tumor detection by iMRI, which were missed by 5-ALA intraoperatively (deemed false negatives). Only 2 studies^14,21^ measured clinical performance as a primary end point, using Karnofsky Performance Scale, European Organisation for Research and Treatment of Cancer (EORTC) Quality of Life Questionnaire (QLQ)-C30, and QLQ-BN20 quality-of-life questionnaires for quantification. Secondary end points included complication rates, overall survival, progression-free survival; 3 studies^21-23^ did not name secondary end points.

All studies used similar protocols for the oral ingestion of 5-ALA (dosing: 20 mg/kg bodyweight), either 2 to 4 hours before induction of anesthesia or 4 to 6 hours before surgery (Table 3). Three studies^21-23^ used Siemens Magnetom 1.5 T iMRI, 1 study used an iMRI device with strength higher than 1.5 T,^14^ while 1 study involved a PoleStar N-20 mobile, ultra-low-field iMRI.^24^ T1-weighted sequences with Gadolinium was mandatory in every study; 3 studies used Magnetization Prepared-RApid Gradient Echo (MPRAGE) sequences,^21-23^ while 2 studies expanded their sequences with T2, fluid-attenuated inversion-recovery, diffusion-weighted imaging, diffusion tensor imaging, and functional imaging.^21,23^

**TABLE 3.** Included Studies and Details of Their Respective Methodologies

Study	5-ALA protocol	iMRI device	iMRI sequences	Extra OR time	Definition of gross total resection	Tumor volumetry	Intended complete resections
Eyüpoglu et al^21^	Oral ingestion of 20 mg/kg bodyweight 3 h before induction of anesthesia	Siemens Magnetom 1.5 T	T1-weighted MPRAGE with contrast, T2-weighted, diffusion-weighted, BOLD functional, diffusion tensor imaging	NS	5-ALA signal no longer detectable, corresponding absence of contrast-enhancing tumor corroborated by iMRI	Tumor volumetry performed immediately before surgery; EoR was calculated as percentage of prior tumor volume using Brainlab	100% of intended complete resections were confirmed by iMRI
Coburger et al^22^	Oral ingestion of 20 mg/kg bodyweight 4 h before surgery	Siemens Magnetom Espree 1.5 T	T1-weighted MPRAGE with contrast	NS	NS	Tumor volumetry performed using Brainlab	NS
Gessler et al^24^	Oral ingestion of 20 mg/kg bodyweight 4-6 h before surgery	PoleStar N-20 mobile ultra-low-field iMRI	T1-weighted with and without contrast	NS	No residual contrast enhancement in early (<72 h) postoperative MRI at 3.0 T	NS	Early postoperative high-field MRI within 72 h after resection confirmed GTR by lack of any remaining contrast-enhancing tissue for the whole cohort except for 1 patient
Coburger et al^23^	Oral ingestion of 20 mg/kg bodyweight 4h before surgery	Siemens Magnetom 1.5 T	T1 MPRAGE with and without Gd-DTPA and an axial T2 and flair sequence	NS	NS	NS	NS
Roder et al^14^	Oral ingestion of 20 mg/kg bodyweight 2-4 h before induction of anesthesia	High-field MRI ≥ 1.5 T	T1-weighted with contrast	Incision-suture times were significantly (*P* < .001) longer in the iMRI arm compared with the 5-ALA arm (316 vs 215 min, respectively)	Residual tumor ≤0.175 cm^3^	Tumor volumetry performed using 3D Slicer	NS

5-ALA, 5-aminolevulinic acid; BOLD, blood-oxygenation-level–dependent; Gd-DTPA: gadolinium diethylenetriaminepentaacetate; iMRI, intraoperative MRI; GTR, gross total resection; MPRAGE, Magnetization Prepared-RApid Gradient Echo; NS, not specified.

Only 1 study reported incision-suture times and correspondingly, if iMRI resulted in longer surgical duration.^14^ Three studies defined GTR as the absence of residual contrast enhancement in MRI, either through iMRI or postoperative MRI^14,21,24^; one defined presence of residual tumor ≤0.175 cm^3^ as GTR.^14^ Tumor volumetry was performed in 3 studies,^14,21,24^ 2 using Brainlab (Feldkirchen, Germany),^21,24^ and 1 using 3D Slicer^14^ softwares.

The EoR, with the goal of GTR, is an important parameter in influencing survival outcomes in patients with HGG.^4,5,25^ Roder et al^14^ demonstrated that GTR was achieved in 81% using iMRI alone compared with 78% with 5-ALA alone, although this difference was not statistically significant (*P* = .790; Figure 2, Table 4). Furthermore, Roder et al^14^ found nonsignificant differences in median overall survival between patients who underwent iMRI alone vs 5-ALA alone (22.9 vs 31.7 months, respectively; *P* = .990 using Cox proportional hazard model adjusted for propensity score).

**TABLE 4.** Included Studies and Their Results and Conclusions

Study	EoR	Limitations of 5-ALA alone	Advantages of iMRI	Limitations of iMRI alone	Clinical performance	Conclusions	Survival
Eyüpoglu et al^21^	• Combined use of 5-ALA and iMRI increased EOR of malignant gliomas located near to eloquent areas from 61.7% to 100% • 5-ALA alone was insufficient to attaining GTR without risk of postoperative deficits	• Efficacy of 5-ALA surgery was dependent on direct visibility of fluorescence, which can be impeded by intervening nonpathological tissue or by blind spots because of angle of vision • 5-ALA does not have the benefit of visualizing directly functional, eloquent areas	• Combination of 5-ALA with iMRI improved EOR from 57.1 (5-ALA alone) to 71.2% in functional grade III (Sawaya) gliomas • In 32.4% the iMRI scan detected residual tumor missed by 5-ALA signal (false impression of complete resection with 5-ALA alone) • Real-time evaluation of EOR using iMRI enables additional cytoreduction in the same operative session • iMRI compensates for potential errors caused by brain shift during surgery • iMRI increases radicality without additional morbidity	• Difficult to precisely delineate tumor margins in real-time becauseiMRI is an offline method requiring pausing resection procedure to acquire images • Vague 5-ALA fluorescence could not be detected by iMRI through contrast enhancement (false negative), representing cellular transformation zones	Karnofsky deterioration of 20% in 1 patient with glioma located adjacent to basal ganglia, otherwise almost all cases had no clinical deterioration with 5-ALA and iMRI	5-ALA and iMRI are complementary modalities in achieving precision and radicality of resections	NS
Coburger et al^22^	NS	• Specificity of 5-ALA (0.43) in detecting solid tumor in HGG is lower than specificity of iMRI (0.70) • 5-ALA shows higher rate of false-positive results for infiltration zone, where early tumor recurrence might arise	Obscured tumor fluorescence might easily be missed without additional iMRI	• Sensitivity is higher in 5-ALA (0.85) compared with iMRI (0.41) • 5-ALA correlated with final histological diagnosis significantly, whereas iMRI had nonsignificant correlation • 5-ALA correlated with Ki-67 index and vascular proliferations, whereas no correlations were found for iMRI • High number of tumor-positive areas were missed in iMRI (false-negatives)	NS	• Higher detection rate for tumor and infiltration zone of GBM in 5-ALA compared with iMRI; however, study does not postulate 5-ALA alone to be superior to iMRI alone • Combination of 5-ALA and iMRI might increase safety of resection adjacent to eloquent areas and prevent overlooking areas missed by 5-ALA alone	NS
Gessler et al^24^	NS		Sensitivity and specificity of iMRI and 5-ALA to detect remaining tumor tissue were 75% and 100% for iMRI, respectively, and 70% and 100% for 5-ALA, respectively	When iMRI device used has limited field strength and image resolution, iMRI might not detect residual tumor which could be detected by 5-ALA	Complication rate 3/32; study did not compare KPS pre- and postoperatively, also no comparison of KPS between 5-ALA alone and iMRI alone	• Both 5-ALA as well as iMRI are helpful in detecting unintentionally remaining tumor tissue • Both iMRI and 5-ALA were the only indicator of further tumor tissue in 26.3% (iMRI) or 21.1% (5-ALA) of the cases, while the other did not indicate residual tumor, when the surgeon thought to have achieved GTR already	Median OS of 80.7 wk (95% CI, 37.6-123.9 wk) and PFS of 61.3 wk (95% CI, 35-87.6 wk)
Coburger et al^23^	NS	Considering the high precision rates of 5-ALA to rule out most of residual tumor, it could be used initially during the resection to check for residual tumor fluorescence. However, 5-ALA is limited by its inability to detect deep seated residual tumor due to intervening, nonfluorescent, healthy tissue obscuring visualization of residual tumor. Adjuncts like lioUS are needed to overcome this limitation	NS	• Patients with unmethylated promoter had a rate of undetected solid tumor of 46% in iMRI, 25% in lioUS, and 20% in 5-ALA. In patients with methylated promoter, rate of undetected tumor was 39%, 15%, and 0%, respectively. Regardless of methylation status, the percentage of missed tumors was the highest with iMRI compared with other modalities (5-ALA and lioUS) • 5-ALA correlated significantly with classification of specimen, presence of necrosis, and microproliferations, as well as methylated MGMT promoter status. Meanwhile, iMRI had no correlations with histopathological findings • iMRI is not economically viable for most neurosurgical centers worldwide compared with 5-ALA and lioUS	NS	• 5-ALA, iMRI, and lioUS detect infiltrating tumor only to a certain extent • Only 5-ALA showed a significant correlation with histopathological findings	NS
Roder et al^14^	Complete resection (defined as residual tumor ≤0.175 cm^3^) was achieved at higher rates with iMRI (115; 81%) than with 5-ALA (90; 78%)	Postoperative rates of neurologic deficits were significantly higher in the 5-ALA than in the iMRI treatment arm. (56% 5-ALA vs 43% iMRI; *P* < .042). However, it is of note that more patients already had preoperative deficits in the 5-ALA arm than in the iMRI arm	Complete resection (defined as residual tumor ≤0.175 cm^3^) was achieved at higher rates with iMRI (115; 81%) than with 5-ALA (90; 78%)	5-ALA might be economically more feasible for glioblastoma resection than iMRI, considering high acquisition and running costs with significantly longer operating room times of iMRI	• Median OS was 31.7 mo in the 5-ALA and 22.9 mo in the iMRI treatment arm, difference was not significant • Both treatment arms had similar PFS and OS	Superiority of iMRI over 5-ALA for achieving complete resections could not be confirmed	Median PFS and OS were comparable in both arms

5-ALA, 5-aminolevulinic acid; EoR, extent of resection; GBM, glioblastoma multiforme; GTR, gross total resection; HGG, high-grade glioma; iMRI, intraoperative MRI; KPS, Karnofsky performance score; lioUS, inear array intraoperative ultrasound; MGMT, methylguanine-DNA-methyltransferase; NS, not specified; OS, overall survival; PFS, progression-free survival.

Eyüpoglu et al^21^ found that the combination of iMRI and 5-ALA significantly improved EoR of HGG adjacent to eloquent areas from 61.7% with 5-ALA alone, to 100% using both 5-ALA and iMRI (*P* < .002; Figure 2). In functional grade III (Sawaya)^26^ gliomas, ie located in eloquent regions, EoR was significantly improved from 57.1% using 5-ALA alone to 71.2% with combined use of 5-ALA and iMRI (*P* < .0003).^21^ Three studies did not report on results regarding EoR.^22-24^ These results suggest that the addition of iMRI to 5-ALA protocols may be superior to 5-ALA alone in improving EoR.

### Limitations of 5-ALA Alone

Eyüpoglu et al^21^ found that attaining GTR with intraoperative use of 5-ALA alone could risk postoperative deficits in functional grade III (Sawaya) gliomas, whereby GTR was achieved in 57.6% with 5-ALA alone vs in 71.2% with 5-ALA and iMRI (*P* < .0003, Table 4). This is corroborated by results of Roder et al,^14^ where use of 5-ALA alone was significantly associated with a higher risk of postoperative deficits (56%) compared with use of iMRI (43%; *P* = .042). This may be confounded by the fact that more patients undergoing intraoperative 5-ALA already exhibited preoperative deficits than those undergoing iMRI.

Coburger et al also found that 5-ALA alone had a lower specificity (0.43) in detecting residual tumor, compared with iMRI (0.70, *P* < .001 using McNemar test),^22^ which could not be corroborated by results found by Gessler et al^24^ (100% with iMRI and with 5-ALA, respectively, *P* not significant; Figure 3). 5-ALA had higher false-positive rates at the infiltration zone, where early tumor recurrence occurs, compared with iMRI. This may explain the increased risk of postoperative deficits using 5-ALA alone, especially when the balance of EoR vs neurological deficits is skewed toward the former based on individual surgeon preference.

### Limitations of iMRI Alone

Coburger et al found that iMRI resulted in lower sensitivity (0.41) in detecting residual tumor compared with sensitivity using 5-ALA (0.85; *P* < .001),^22^ conflicting with the results of the study by Gessler et al (75% with iMRI vs 70% with 5-ALA, P not significant; Figure 4).^24^ iMRI did not demonstrate a significant correlation with results of histopathological confirmation including Ki-67 index and vascular proliferation (*P* = .112 using Spearman rho test), whereas a significant correlation could be demonstrated with 5-ALA (*P* = .032). Independent of methylation status of patients, use of iMRI was associated with higher rates of false-negative ie missed tumor sites, compared with 5-ALA, or even to lioUS. Higher false-negative rates may ultimately result in lower rates of GTR. Gessler et al^24^ demonstrated that lower field strength <1.5 T might compromise tumor detection rates by iMRI compared with 5-ALA.

## DISCUSSION

The solitary use of either 5-ALA or iMRI in maximizing EoR in HGG surgery is hindered by significant limitations. Eyüpoglu et al^21^ and Coburger et al^22^ attributed the main limitation of 5-ALA to its dependency on direct visualization of fluorescence, which may be obscured by intervening healthy parenchyma or blood inside the intraoperative site. Previous studies have found that 5-ALA fluorescence was not specific to neoplastic cells in glioblastoma,^27,28^ as nonneoplastic cells displayed 5-ALA fluorescence within the tumor microenvironment.^27,28^ Moreover, unlike MRI, 5-ALA lacks the ability to show anatomic regions and spatial relationships. Eyüpoglu et al^21^ found that iMRI was able to detect 32.4% of residual tumor missed by 5-ALA alone intraoperatively. Similarly, Coburger et al^22^ postulates that combined use of 5-ALA and iMRI allows iMRI to detect residual tumor missed by 5-ALA alone, while increasing safety of resection by delineating eloquent areas. Gessler et al^24^ found that either iMRI or 5-ALA was the only indicator of residual tumor tissue in 26.3% (iMRI) or 21.1% (5-ALA) of the cases where attainment of GTR was assumed by the neurosurgeon. Thus, the synergistic effects of the combined use of 5-ALA and iMRI may increase the extent of tumor resection.

Residual tumor adjacent to eloquent areas detected by 5-ALA with real-time visualization may be corroborated with iMRI, superimposed preoperative tractography and/or electrophysiological neuromonitoring, or with adjuncts such as lioUS,^23^ to prevent damage to eloquent structures. Application of iMRI after presumed GTR by the neurosurgeon, with no residual fluorescence visualized by 5-ALA, allows detection of nonfluorescent tumor corresponding to contrast-enhancing areas. In this way, iMRI enables additional cytoreduction within the same surgery, sparing patients re-resection because of residual tumor being detected only in postoperative MRI. Thus, Eyüpoglu et al^21^ argues that additional iMRI increases tumor EoR without the additional morbidity risk with 5-ALA alone. Furthermore, iMRI corrects for brain shifts caused by the resection of space-occupying tumors, compared with 5-ALA with neuronavigation using nonupdated, preoperative MRI images.

The studies in this review eludicated on various limitations of solitary use of iMRI. Eyüpoglu et al^21^ argues that iMRI is not a real-time, intraoperative visualization in a way that 5-ALA is because the offline nature of iMRI image acquisition necessitates a temporary interruption of tumor resection. Areas of discrete 5-ALA fluorescence, representing cellular transformation zones, may yield false-negative results with iMRI. Roder at al^14^ argues that higher costs (eg device acquisition, electricity) and significantly longer surgeries render iMRI economically disadvantageous compared with 5-ALA, especially in developing countries unable to afford iMRI. These limitations of iMRI may explain why it has not yet developed as a standalone tool in detecting residual tumor during resection surgery.

The results of our systematic review and meta-analysis suggest that the combination of 5-ALA and iMRI improves EoR and intraoperative residual tumor detection rates, compared with use of either modality alone. These confirm results of previous studies investigating the combined use of 5-ALA and iMRI.^17,29-31^ One study provided contradicting results; however, it used a low-field iMRI device (PoleStar N30),^32^ which Gessler et al^24^ found to be associated with lower sensitivity and specificity. The results of our review including only prospective studies corroborate the findings of an earlier meta-analysis by Golub et al^17^ that neither 5-ALA nor iMRI demonstrated superior GTR rates compared with the other.

The 5 studies included in this review were prospective, nonrandomized studies, one of which is a multicentre clinical trial.^14^ Four studies showed low-to-moderate risk of bias according to the ROBINS-I assessment, with 1 study displaying a moderate risk of bias (Table 5). The singular nonrandomized controlled trial had a low Jadad score. Furthermore, data extraction revealed numerous parameters which some studies did not provide or specify. This limited the meta-analysis of EoR and tumor detection sensitivity and specificity, which were not consistently investigated across all 5 studies. Thus, the heterogeneity of these studies and their designs introduces potential bias and confounding factors to the results of this review.

**TABLE 5.** Included Studies and ROBINS-I Risks of Bias Assessment

Study	Country	Confounding bias in selection of participants	Bias in classification of interventions	Bias due to deviations from intended interventions	Bias due to missing data	Bias in measurement of outcomes	Bias in selection of the reported result	Overall risk of bias judgment
Eyüpoglu et al^21^	Germany	Moderate	Low to moderate	Low	Moderate	Low to moderate	Low	Moderate
Coburger et al^22^	Germany	Moderate	Low	Low	Moderate	Moderate	Low	Low to moderate
Gessler et al^24^	Germany	Moderate	Low	Low	Low	Low	Low	Low
Coburger et al^23^	Germany	Moderate	Low	Low	Moderate	Low to moderate	Low	Low to moderate
Roder et al^14^	Germany	Moderate	Low	Low	Low	Low	Low	Low

Our meta-analysis was impeded by the relative low patient numbers in the 4 prospective studies compared with the 314 patients in the nonrandomized controlled trial,^14^ which makes up almost 70% of all the patients included in the 5 studies and therefore can be considered to carry more weight of evidence. To avoid introducing bias, our meta-analysis did not include pooled data from all the patients in the 5 studies.

Previous systematic reviews and meta-analyses on this topic analyzed data before 2011^12^ and retrospective studies not comparing iMRI with 5-ALA^13^ directly.^17^ To our knowledge, this is the first systematic review and meta-analysis investigating the advantages of iMRI over 5-ALA in resection surgery of HGG, including only prospective studies and a nonrandomized controlled trial comparing both modalities directly.

For ethical reasons, randomization of patients with HGG into comparison arms 5-ALA alone vs iMRI alone is unlikely to be feasible, especially in consideration of our results suggesting that the combined use of 5-ALA and iMRI may be superior to using either modality alone intraoperatively, which is corroborated by previous studies.^12,13^

Nevertheless, our findings question the assumption that iMRI alone is superior to 5-ALA alone intraoperatively in maximizing EoR and thus improving outcomes significantly in patients with HGG. Indeed, the results of this review imply that, whenever economically feasible and with iMRI devices ≥1.5 T, addition of iMRI to conventional resection surgery with 5-ALA may be more advantageous compared with iMRI alone or 5-ALA alone because the synergistic combination of both modalities allows them to compensate for each other's limitations. Our results contribute to guiding current surgical practices of neurosurgeons in resection surgery of HGG. Future research is warranted with larger prospective comparisons of these techniques and investigating associations between EoR and underlying molecular features of glioblastoma.^33,34^

## CONCLUSION

This study systematically reviewed prospective studies directly comparing use of 5-ALA vs iMRI in resection surgery of adult patients with HGG and conducted a meta-analysis of sensitivity, specificity, and EoR. The results suggest iMRI alone is not superior to 5-ALA alone in attaining GTR and achieving improved outcomes in this population. Combined use of 5-ALA and iMRI appears to increase detection of residual tumor intraoperatively, which improves EoR compared with 5-ALA alone or iMRI alone, without compromising postoperative neurologic function. Future results of ongoing RCTs on this topic are needed to confirm these findings.

## Supplemental Digital Content

**Supplemental Digital Content 1.** Research Reporting Guideline Checklist—PRISMA 2020 Checklist. **Supplemental Digital Content 2.** Database searches employed synonyms of the terms “5-ALA,” “iMRI,” “glioblastoma,” “high-grade glioma,” “resection surgery” and “randomized clinical trial” in PubMed, Embase, Scopus, and ClinicalTrials.gov.
